# Elevated angiotensin II induces platelet apoptosis through promoting oxidative stress in an AT1R‐dependent manner during sepsis

**DOI:** 10.1111/jcmm.16382

**Published:** 2021-02-23

**Authors:** Dun‐Feng Xu, Yu‐Jian Liu, Yan‐Fei Mao, Yan Wang, Chu‐Fan Xu, Xiao‐Yan Zhu, Lai Jiang

**Affiliations:** ^1^ Department of Anesthesiology and Surgical Intensive Care Unit Xinhua Hospital Shanghai Jiao Tong University School of Medicine Shanghai China; ^2^ School of Kinesiology The Key Laboratory of Exercise and Health Sciences of Ministry of Education Shanghai University of Sport Shanghai China; ^3^ Department of Physiology Navy Medical University Shanghai China

**Keywords:** angiotensin II (Ang II), angiotensin II receptor blocker (ARB), apoptosis, platelet, reactive oxygen species (ROS), thrombocytopenia

## Abstract

Thrombocytopenia is independently related with increased mortality in severe septic patients. Renin‐angiotensin system (RAS) is elevated in septic subjects; accumulating studies show that angiotensin II (Ang II) stimulate the intrinsic apoptosis pathway by promoting reactive oxygen species (ROS) production. However, the mechanisms underlying the relationship of platelet apoptosis and RAS system in sepsis have not been fully elucidated. The present study aimed to elucidate whether the RAS was involved in the pathogenesis of sepsis‐associated thrombocytopenia and explore the underlying mechanisms. We found that elevated plasma Ang II was associated with decreased platelet count in both patients with sepsis and experimental animals exposed to lipopolysaccharide (LPS). Besides, Ang II treatment induced platelet apoptosis in a concentration‐dependent manner in primary isolated platelets, which was blocked by angiotensin II type 1 receptor (AT1R) antagonist losartan, but not by angiotensin II type 2 receptor (AT2R) antagonist PD123319. Moreover, inhibiting AT1R by losartan attenuated LPS‐induced platelet apoptosis and alleviated sepsis‐associated thrombocytopenia. Furthermore, Ang II treatment induced oxidative stress level in a concentration‐dependent manner in primary isolated platelets, which was partially reversed by the AT1R antagonist losartan. The present study demonstrated that elevated Ang II directly stimulated platelet apoptosis through promoting oxidative stress in an AT1R‐dependent manner in sepsis‐associated thrombocytopenia. The results would helpful for understanding the role of RAS system in sepsis‐associated thrombocytopenia.

## 1 INTRODUCTION

Sepsis describes the life‐threatening systemic inflammatory state and organ dysfunction caused by a dysregulated host response to infection.^1^ Evidence is growing that platelets are important blood elements responsible for homeostasis maintenance and fighting infections.^2^, ^3^ Platelets contain and release a large number of immune molecules that directly impact the development and resolution of inflammation.^4^, ^5^ The changes of quality and quantity of platelets are closely associated with the morbidity and mortality of infectious diseases.^2^, ^4^, ^6^ As a frequent laboratory abnormality in patients with sepsis, thrombocytopenia is found to be independently related to increased mortality in severe sepsis patients.^2^, ^7^ Hence, rectification of thrombocytopenia is needed to avoid the potentially lethal complications of severe sepsis.

Although the consumption of platelets has been used to interpret sepsis‐associated thrombocytopenia frequently,^8^ evidence of platelet apoptosis has also been found during sepsis, such as increased apoptotic vesicles and surface activation markers.^9^ Peptidoglycan, a component of bacterial cell wall, triggers apoptosis of platelets by showing cell membrane scrambling, activated caspase‐3 and depolarized mitochondria.^10^ Moreover, *Escherichia coli* and Staphylococcus bacterial isolated from the septic patients can directly trigger the process of intrinsic apoptotic cell death in platelets in vitro.^11^, ^12^ However, the mechanisms underlying platelet apoptosis in sepsis have not been fully elucidated.

The renin‐angiotensin system (RAS) has been implicated in preventing systemic hypotension during the development of septic shock.^13^, ^14^ Furthermore, angiotensin II (Ang II) has been recognized as a key player in several biological processes, including coagulation, apoptosis and inflammatory response.^15^, ^16^ Doerschug et al^17^ have reported that plasma Ang II is elevated in septic patients as compared to volunteers. Moreover, the level of Ang II elevation correlates with organ failure and with measures of microvascular dysregulation. Notably, accumulating studies in various cell types have demonstrated that Ang II stimulates the intrinsic pathway of apoptotic cell death by promoting intracellular reactive oxygen species (ROS) production.^15^, ^18^, ^19^ Therefore, this study hypothesized that thrombocytopenia in patients with sepsis was associated with increased plasma Ang II and that treatment with angiotensin II receptor blocker (ARB) would attenuate sepsis‐induced platelet apoptosis and improve septic thrombocytopenia in a murine model of lipopolysaccharide (LPS)‐induced endotoxemia.

## 2 MATERIALS AND METHODS

### 2.1 Plasma of healthy volunteers and patients with sepsis

Septic patients were all recruited in Xinhua Hospital from January 2018 to June 2019. Healthy adults that underwent routine physical examinations were recruited as healthy volunteers. More details were provided in Supporting information.

### 2.2 Endotoxemia model and drug treatment

Male, 7‐9‐weeks‐old mice were used in this study. Purified LPS was injected intraperitoneally (i.p.) (5 mg/kg) as described previously.^20^ The angiotensin II type 1 receptor (AT1R) antagonist losartan and ROS scavenger N‐acetyl‐l‐cysteine (NAC) were dissolved in sterile pyrogen‐free saline. Both losartan (10‐30 mg/kg) and NAC (100 mg/kg) were treated i.p. 30 minutes prior to the treatment of LPS. For Kaplan‐Meier survival curve, mice were challenged with a lethal dose of LPS (30 mg/kg) by i.p. injection with or without pre‐treated with losartan i.p. in 30 minutes. Then, mice were monitored carefully for lethality per 6 h for up to 48 hours. More details were provided in the supplementary data.

### 2.3 Measurements of plasma renin activity and Ang II concentration

The plasma renin activity (PRA) and Ang II concentration were measured by Radioimmunoassay Kit. Details were provided in Supporting information.

### 2.4 Platelet counts

Platelet numbers were determined using an automated counter (XS‐500i; Sysmex, Kobe, Japan).

### 2.5 Mouse platelet isolation and treatment

Mouse platelets were isolated by a modified method as previously described.^21^, ^22^ Details were described in Supporting information. For in vitro studies, platelet pellets were resuspended in serum‐free M199 Medium with 5 × 10^7^ cells in each group.^23^ Freshly isolated platelets were then treated with or without Ang II (50, 100 or 200 nmol l^−1^),^24^, ^25^ NAC (5 mmol l^−1^), losartan (10 μmol l^−1^) or PD123319 (10 μmol l^−1^) for 24 hours.

### 2.6 Western blot analysis

The washed platelets were homogenized, and proteins were used to perform Western blot assay. Details were described in the supplementary data.

### 2.7 Measurement of Caspase‐3 colorimetric proteolytic activity

Caspase‐3 activity was determined by the Caspase 3 Activity Assay Kit. Details were described in Supporting information.

### 2.8 Detection of ROS

Intracellular ROS levels were measured with fluorescence probe 2′,7′‐dichlorofluorescein diacetate. Details were described in Supporting information.

### 2.9 Detection of malondialdehyde

Intracellular malondialdehyde (MDA) levels were measured as previously described.^26^ Details were described in Supporting information

### 2.10 Measurements of H_2_O_2_ and glutathione peroxidase activity

Platelets were washed and homogenized in cold assay buffers. H_2_O_2_ content and glutathione peroxidase (GPx) activity in platelets were determined by H_2_O_2_ detection kit and GPx assay kit according to manufacturer's instructions, respectively.

### 2.11 Statistical analysis

All data are expressed as means ± standard deviation (SD). Statistical comparisons between two groups were determined by two‐tailed Student's *t* test. One‐way ANOVA with Tukey's post hoc test and non‐parametric statistical tests (Kruskal‐Wallis) was performed for comparisons among multiple groups. Pearson's correlation was used to examine the relationship between platelet count and PRA or Ang II. To determine statistical significance between survival curves, Kaplan‐Meier test was used. All statistical analyses were performed with SPSS 16.0 (SPSS Inc., Chicago, USA). A value of *P* <0.05 was considered significant.

## 3 RESULTS

### 3.1 Plasma Ang II correlates inversely with platelet count in septic patients

**FIGURE 1:**
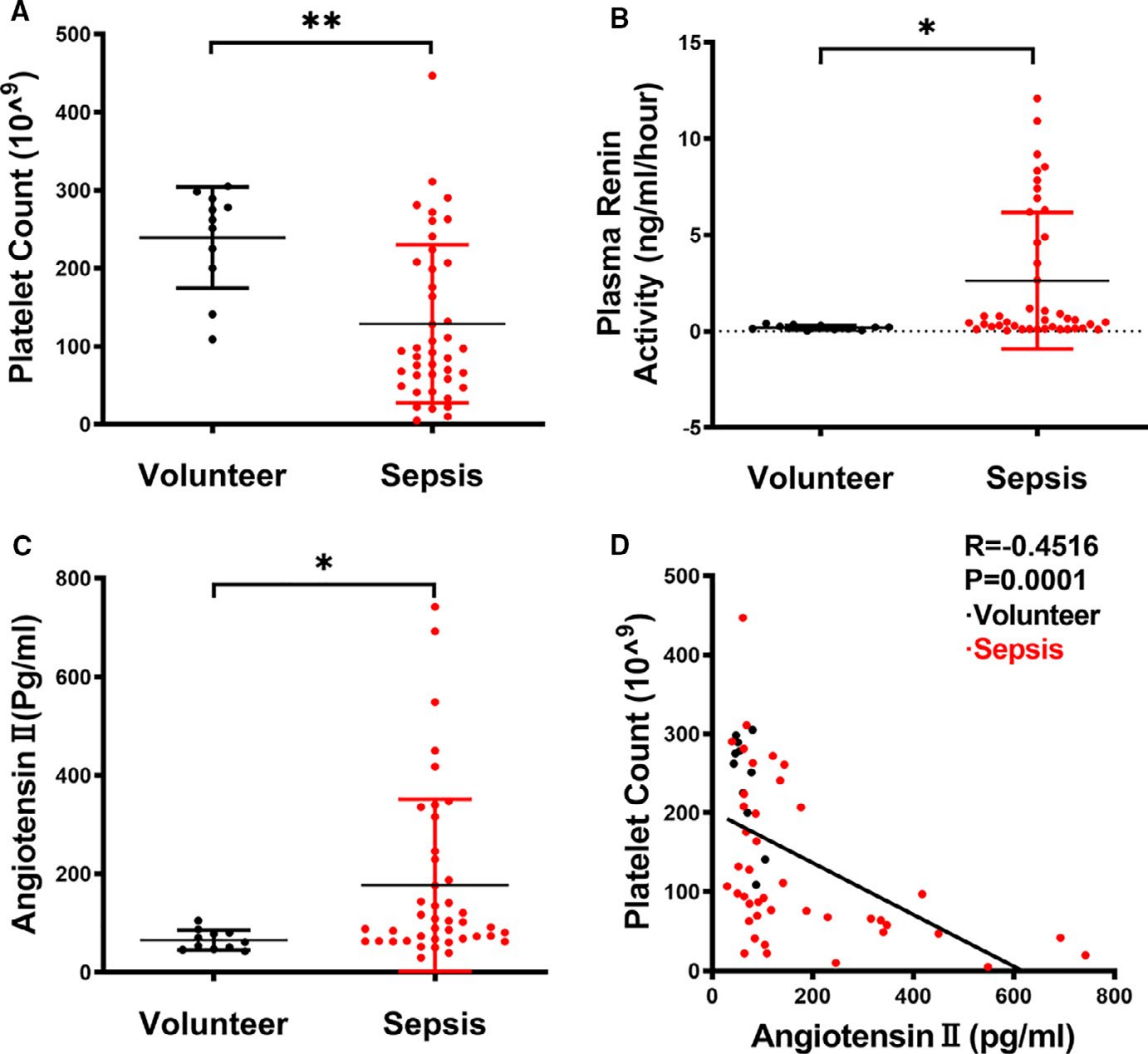
Plasma Ang II correlates inversely with platelet count in patients with sepsis. Platelet count (A), plasma renin activity (B) and plasma Ang II (C) levels in septic patients (n = 42) and healthy volunteers (n = 11). (D) Correlation analysis between plasma Ang II levels and platelet count. *P <0.05, **P <0.01

This study recruited a total of 42 septic patients fulfilling our enrolment criteria and 11 healthy volunteers. The general characteristics of the patients with sepsis were summarized in Table 1. The subjects showed an age range from 37 to 85 years, and a slight male predominance (Male: female ratio 2.23:1). The primary diagnosis of the patients with sepsis included gastric carcinoma, colorectal cancer, choledocholithiasis, gastrointestinal, pancreatic cancer, hepatoma, urinaemia, multiple fracture, urinary tract infection, gallbladder carcinoma and duodenal papilla carcinoma. As shown in Figure 1A‐C, average values for the platelet count (129 × 10^9^/L, range 5 × 10^9^/L to 447 × 10^9^/L) were significantly decreased, whereas average values for PRA (2.63 ng/mL/h, range 0.05 ng/mL/h to 12.08 ng/mL/h) and Ang II (176.83 Pg/mL, range 29.57‐742.13 Pg/mL) were significantly elevated in septic patients as compared with healthy volunteers. Notably, plasma Ang II levels inversely correlated with the platelet count (*r* = −0.4516, *P* =0.0001; Figure 1D).

**TABLE 1:** Clinical patient characteristics

	Sepsis	Volunteers
Number of patients	42	11
Gender (male/female)	29/13	4/7
Age (years)	61 (37‐85)	42 (25‐59)
Platelet counts (×10^9^)	129 (5‐447)	239 (109‐305)
Severity score		
APACHE II	21 (3‐39)	
MODS	10 (1‐19)	
SOFA	16 (1‐31)	
Primary diagnosis		
Gastric carcinoma	9	
Colon cancer	7	
Choledocholithiasis	1	
Gastrointestinal	9	
Pancreatic cancer	3	
Hepatoma	1	
Urinaemia	1	
Multiple fracture	1	
Urinary tract infection	8	
Gallbladder carcinoma	1	
Duodenal papilla carcinoma	1	
Site of infection		
Pulmonary	5	
Abdominal	27	
Urinary	10	
Biliary tract Infection	1	

Note

Numbers are given as median and (range).

Abbreviations: APACHE II, Acute Physiology and Chronic Health Evaluation II; MODS, Multiple Organ Dysfunction Score; SOFA, Sequential Organ Failure Assessment.

### 3.2 Elevated plasma Ang II is associated with platelet apoptosis and thrombocytopenia in LPS‐induced endotoxemia mice

**FIGURE 2:**
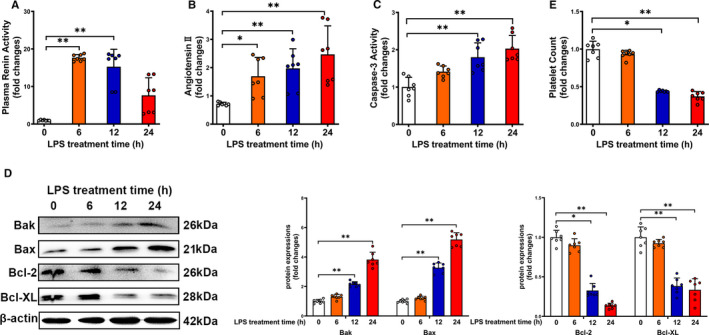
Elevated plasma Ang II is associated with platelet apoptosis and thrombocytopenia in LPS‐induced endotoxemia mice. Plasma renin activity (A), plasma Ang II levels (B), platelet caspase‐3 activity (C), expressions of Bak, Bax, Bcl‐2 and Bcl‐XL in platelets (D) and platelet count (E) (n = 7). *P < 0.05, **P < 0.01. kDa, kiloDalton

We then detected PRA and Ang II level in mice injected with LPS for various time‐points. As shown in Figure 2A, PRA peaked at 6 hours and progressively declined at 12 and 24 hours after LPS administration. Plasma Ang II level was elevated from 6 to 24 hours after LPS administration, indicating the persistent increase of Ang II‐induced by LPS (Figure 2B). Evidence of platelet apoptosis has been shown during the pathogenesis of sepsis.^9^ We found that LPS treatment for 12‐24 hours significantly increased caspase‐3 activity in platelets (Figure 2C). As shown in Figure 2D, pro‐apoptotic proteins (Bak and Bax) were increased, whereas anti‐apoptotic proteins (Bcl‐2 and Bcl‐XL) were decreased from 12 to 24 hours after LPS administration. Furthermore, platelet counts were profoundly decreased at 12 and 24 hours after LPS administration (Figure 2E). These findings indicated that elevated plasma Ang II is associated with platelet apoptosis and thrombocytopenia in LPS‐treated mice.

### 3.3 Ang II treatment induces platelet apoptosis in a concentration‐dependent manner in primary isolated platelets

**FIGURE 3:**
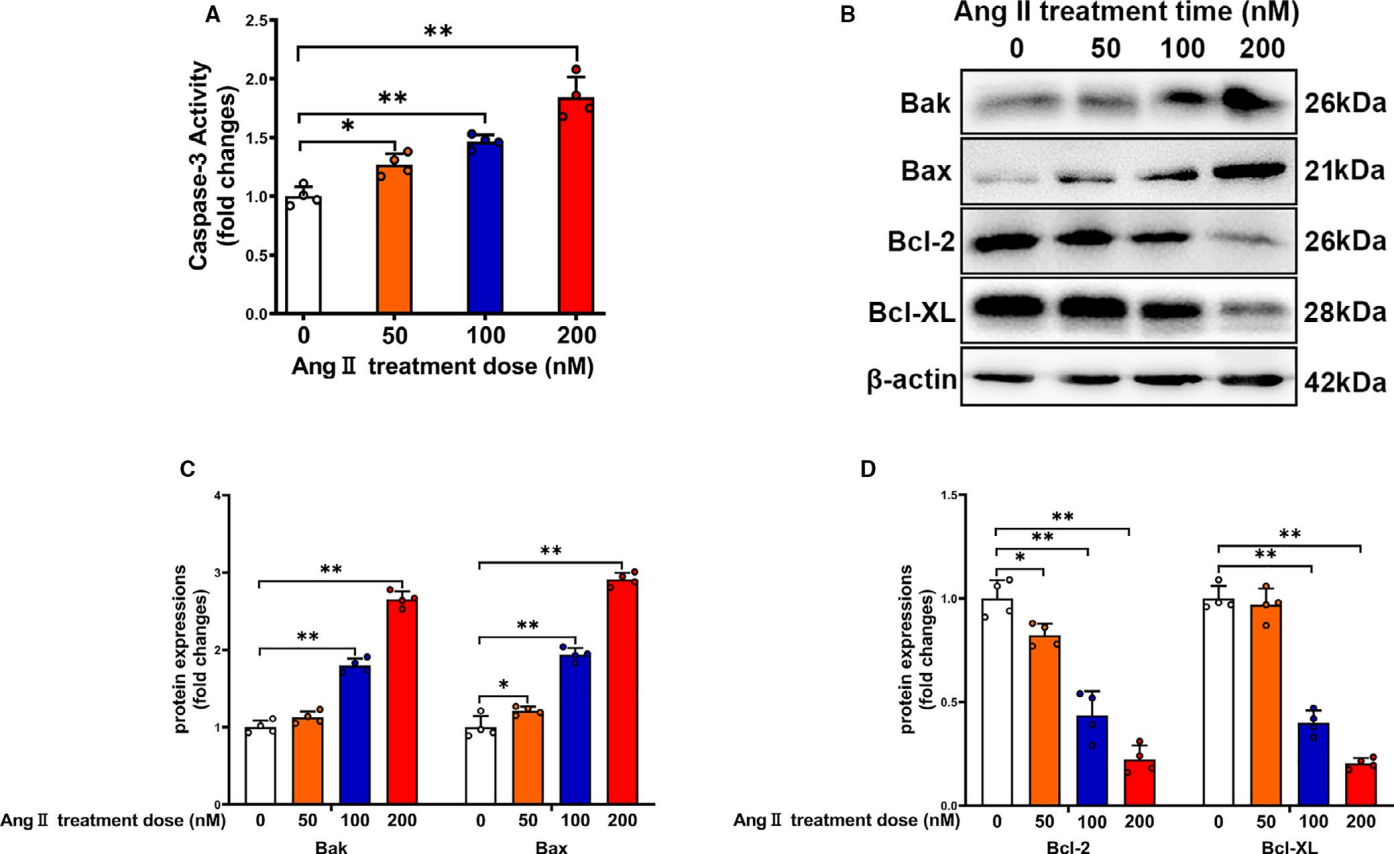
Ang II treatment leads to platelet apoptosis in a concentration‐dependent manner in primary isolated platelets. Primary isolated mouse platelets were incubated with Ang II for 24 h. Caspase‐3 activity (A), expressions of Bak, Bax, Bcl‐2 and Bcl‐XL (B‐D) were determined in platelets (n = 4). *P < 0.05, **P <0.01. kDa, kiloDalton

To directly explore whether the elevated RAS was involved in the pathogenesis of sepsis‐associated thrombocytopenia, we used different gradient concentrations of Ang II to stimulate primary isolated mouse platelets. As shown in Figure 3A, Ang II treatment significantly increased caspase‐3 activity in platelets in a concentration‐dependent manner. In addition, the Ang II induced accumulation of pro‐apoptotic proteins (Bak and Bax) and decreased the anti‐apoptotic proteins (Bcl‐2 and Bcl‐XL) expression in a concentration‐dependent manner (Figure 3B‐D). Together, the data indicated that Ang II induced apoptotic activity in primary isolated platelets.

### 3.4 Ang II treatment leads to increased oxidative stress level in a concentration‐dependent manner in primary isolated platelets

**FIGURE 4:**
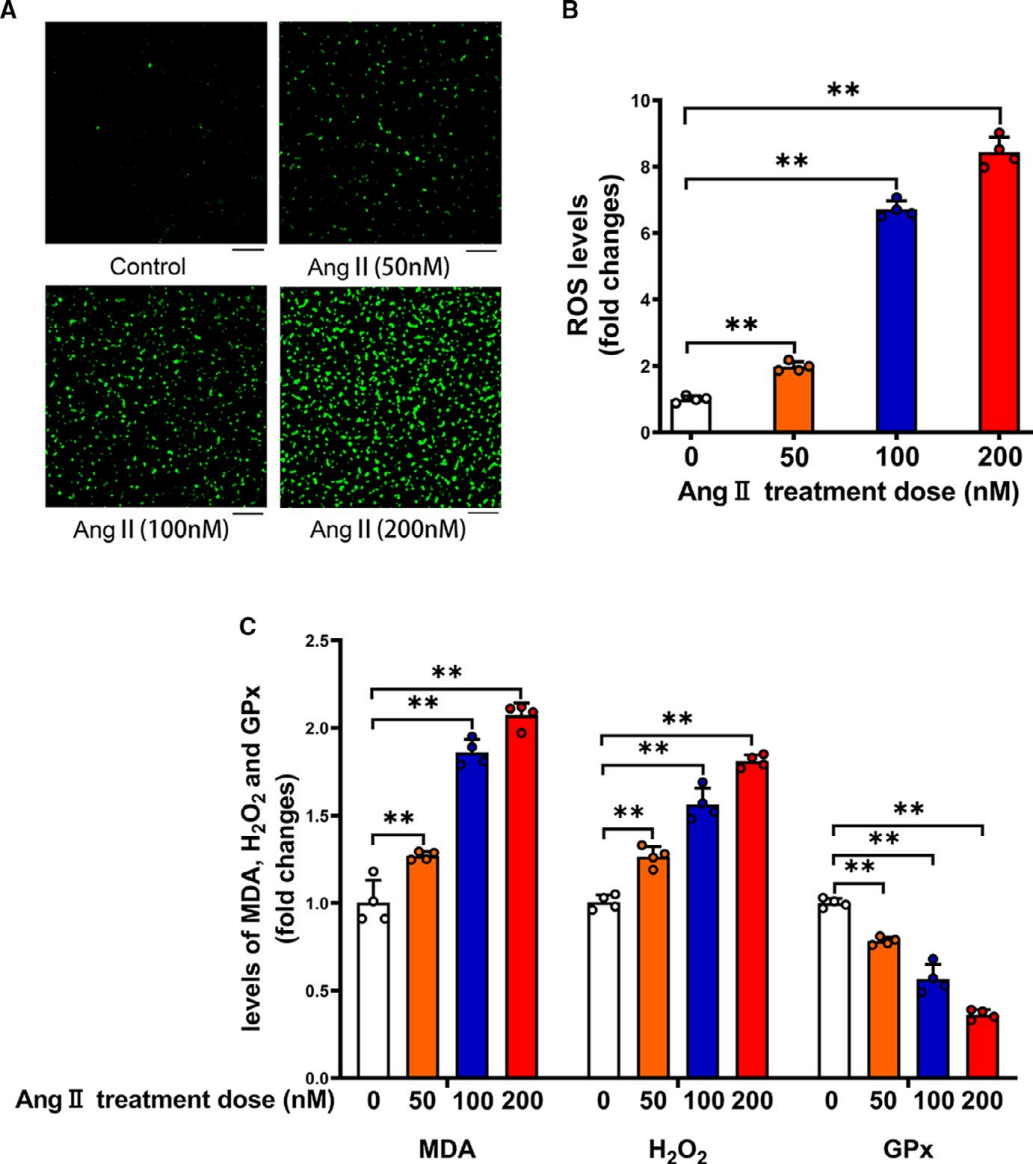
Ang II treatment induces oxidative stress level in a concentration‐dependent manner in primary isolated platelets. Primary isolated mouse platelets were incubated with Ang II for 24 h. ROS production (A&B), levels of MDA, H_2_O_2_ and GPx activity (C) were determined in platelets (n = 4). Scale bars = 50 μm. **P < 0.01. kDa, kiloDalton

Ang II is known to activate intrinsic apoptosis pathway by promoting ROS production in various cell types.^15^, ^27^, ^28^ By measuring the fluorescence of DCFH‐DA over a 30 minutes incubation period, we found that ROS production was increased significantly after treatment of platelets with Ang II in a concentration‐dependent manner in primary isolated platelets (Figure 4A,B). We then determined two pro‐oxidant biomarkers MDA and H_2_O_2_. As shown in Figure 4C, MDA and H_2_O_2_ levels were significantly elevated in Ang II‐treated platelets compared to control platelets in a concentration‐dependent manner. In contrast, activity of the antioxidant enzyme GPx in platelets was significantly decreased by Ang II treatment. All data showed that elevated oxidative stress may be involved in Ang II‐related pathogenesis of sepsis‐associated thrombocytopenia.

### 3.5 Inhibiting oxidative stress by ROS scavenger NAC suppresses the Ang II‐induced platelet apoptosis

**FIGURE 5:**
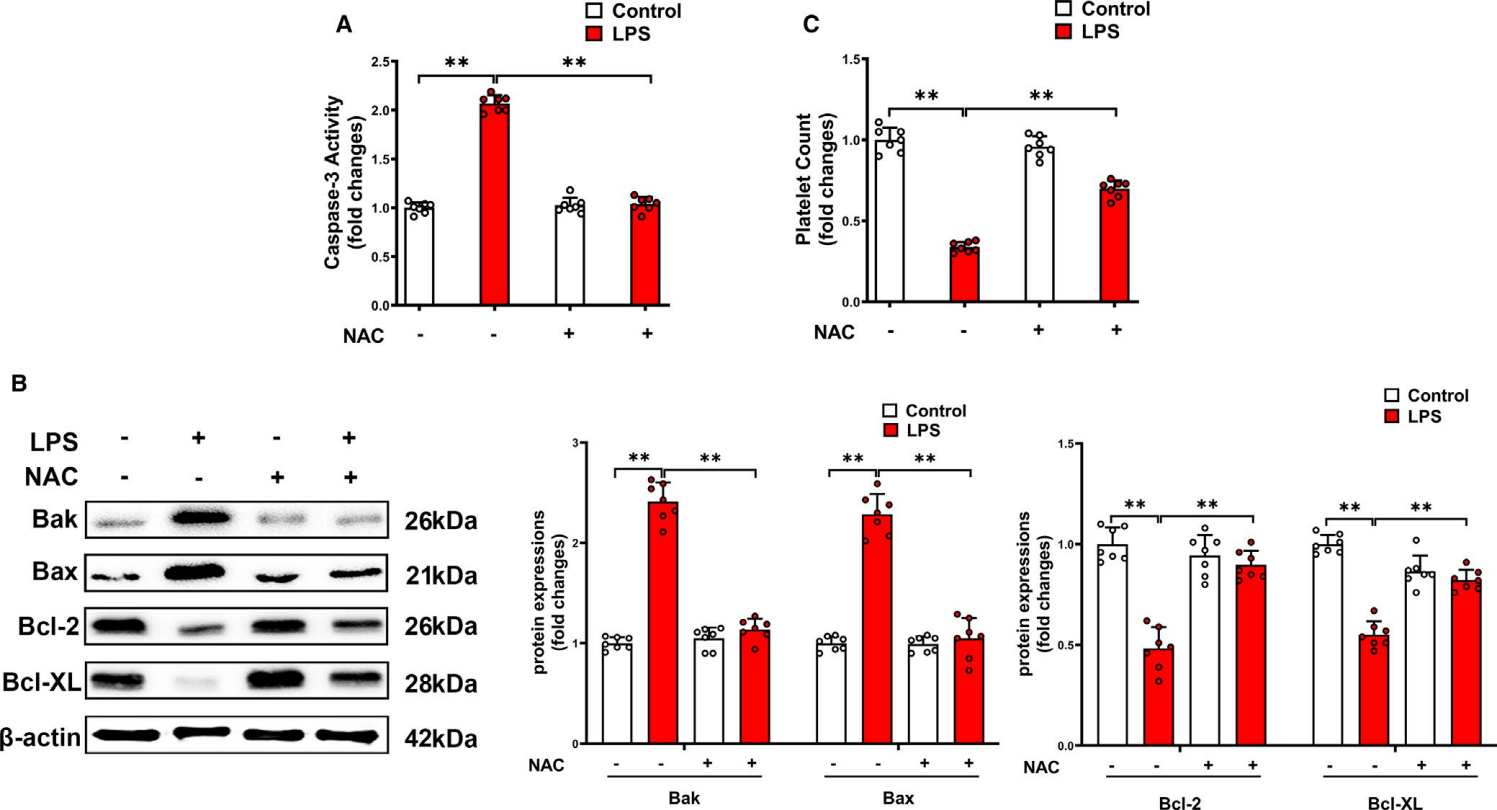
The effect of ROS scavenger NAC on platelet apoptosis and thrombocytopenia in LPS‐induced endotoxemia mice. Twenty‐four hours after NAC (100 mg/kg) treatment, caspase‐3 activity (A), expressions of Bak, Bax, Bcl‐2 and Bcl‐XL (B) in platelets, and platelet count (C) were determined (n = 7). **P < 0.01. kDa, kiloDalton

**FIGURE 6:**
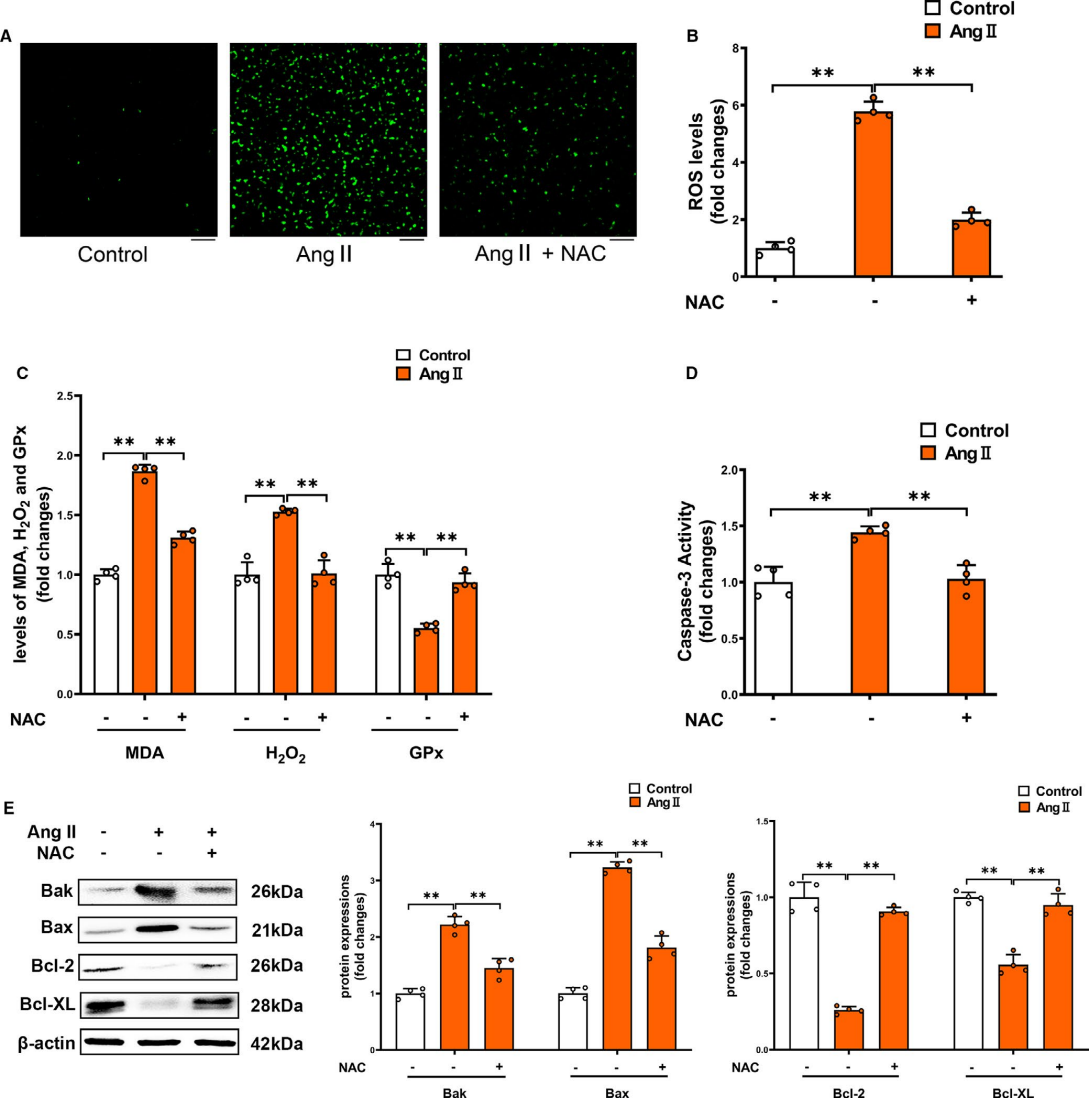
The effect of ROS scavenger NAC on Ang II‐induced oxidative stress and platelet apoptosis in primary isolated platelets. Primary isolated mouse platelets were incubated with 100 nmol l^−1^ Ang II and/or 5 mmol l^−1^ NAC for 24 h. ROS production (A&B), levels of MDA, H_2_O_2_ and GPx activity (C), caspase‐3 activity (D), and expressions of Bak, Bax, Bcl‐2 and Bcl‐XL (E) were determined in platelets (n = 4). Scale bars = 50 μm. **P < 0.01. kDa, kiloDalton

To further explore that whether the oxidative stress is involved in Ang II‐related pathogenesis in sepsis‐associated thrombocytopenia, we used ROS scavenger NAC to treat LPS‐induced endotoxemia mice model. We found that ROS scavenger NAC attenuated platelet caspase‐3 activity (Figure 5A), decreased the pro‐apoptotic proteins (Bak and Bax) and reversed the loss of anti‐apoptotic proteins (Bcl‐2 and Bcl‐XL) (Figure 5B), leading to improvement of thrombocytopenia (Figure 5C). So we used the NAC to treat Ang II‐induced primary isolated platelets. NAC entirely blocked the ROS production via inhibition of two pro‐oxidant biomarkers (MDA and H_2_O_2_) and improvement of activity of the antioxidant enzyme GPx (Figure 6A‐C). Furthermore, NAC blocked the Ang II‐induced caspase‐3 activation in platelets (Figure 6D). The Ang II‐induced accumulation of Bak and Bax was largely decreased, and the loss of Bcl‐2 and Bcl‐XL was dramatically prevented by NAC (Figure 6E). Our findings indicated that Ang II directly promotes platelet ROS production and oxidative stress, thus leading to platelet apoptosis.

### 3.6 Ang II treatment leads to platelet apoptosis in primary isolated platelets in an AT1R‐dependent manner

**FIGURE 7:**
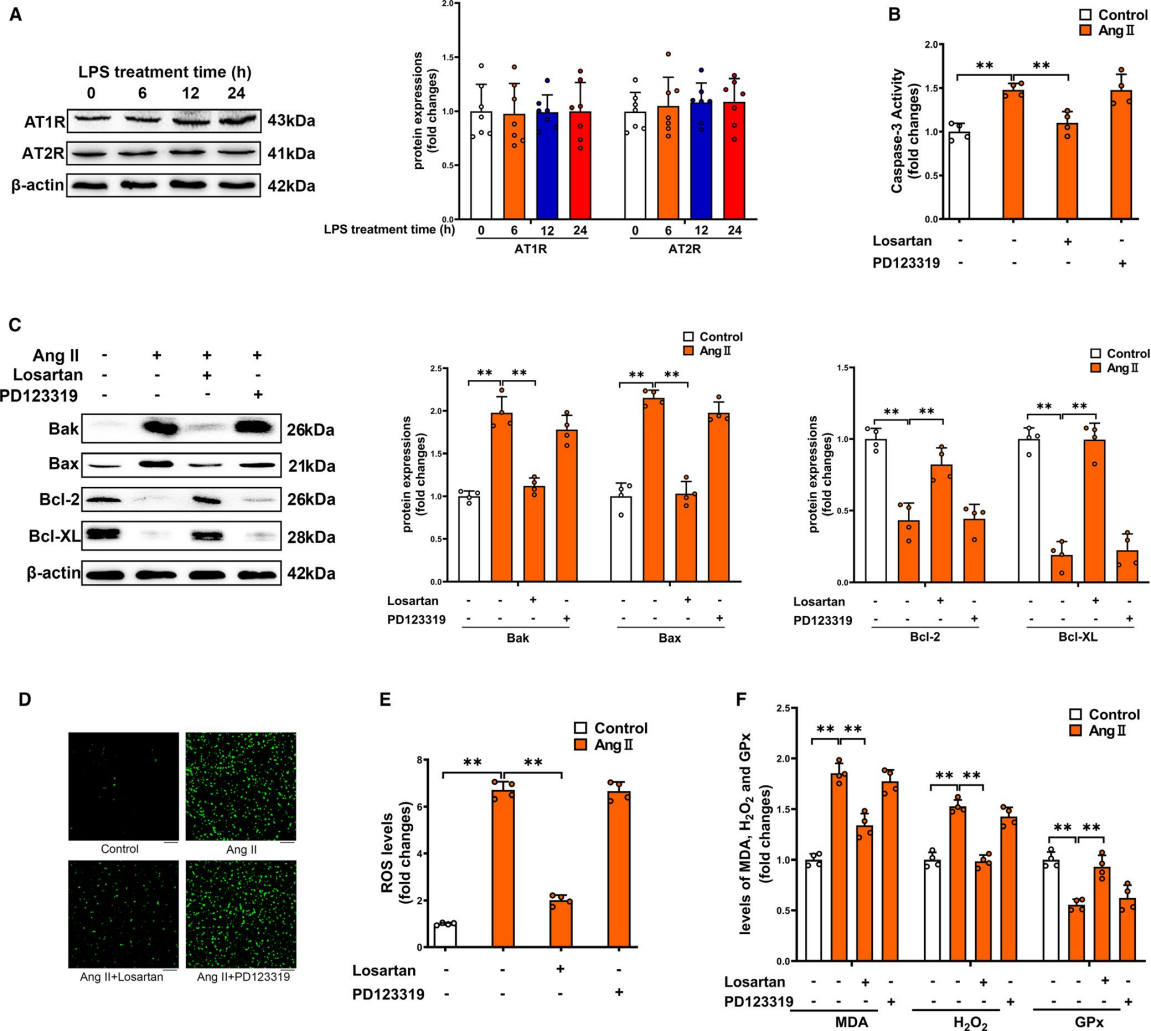
Ang II treatment leads to platelet apoptosis through promoting oxidative stress in primary isolated platelets in an AT1R‐dependent manner. Protein levels of AT1R and AT2R (A) in platelet obtained from control and LPS‐treated mice were determined. Primary isolated mouse platelets were incubated with 100 nmol l^−1^ Ang II, 10 μmol l^−1^ losartan, 10 μmol l^−1^ PD123319 for 24 h. Caspase‐3 activity (B), expressions of Bak, Bax, Bcl‐2 and Bcl‐XL (C), ROS production (D&E), levels of MDA, H_2_O_2_ and GPx activity (F) were determined in platelets (n = 4). Scale bars = 50 μm. **P < 0.01. kDa, kiloDalton

Two Ang II receptors, designated AT1R and AT2R, mediate the biological functions of Ang II. Both AT1R and AT2R are expressed in platelets.^29^, ^30^ We found that LPS administration did not affect protein expressions of AT1R and AT2R in platelets at 6‐24 hours after the administration (Figure 7A). To determine whether Ang II could directly act on platelets, primary isolated mouse platelets were used. As shown in Figure 7B, Ang II treatment significantly increased caspase‐3 activity in platelets. AT1R antagonist losartan blocked the Ang II‐induced caspase‐3 activation in platelets, which was not affected by AT2R antagonist PD123319. In addition, the Ang II‐induced accumulation of Bak and Bax was largely decreased, whereas the loss of Bcl‐2 and Bcl‐XL was dramatically prevented by losartan (Figure 7C). AT2R antagonist PD123319 had no significant effect on pro‐apoptotic or anti‐apoptotic proteins in Ang II‐treated platelets. Taken together, our results demonstrated that Ang II directly stimulated platelet apoptosis in an AT1R‐dependent manner. In addition, losartan blocked Ang II‐induced ROS production in platelets, which was not affected by PD123319 (Figure 7D,E). As shown in Figure 7F, MDA and H_2_O_2_ levels were significantly elevated in Ang II‐treated platelets compared to control platelets. In contrast, activity of the antioxidant enzyme GPx in platelets was significantly decreased by Ang II treatment, which was partially reversed by losartan. All results showed that Ang II treatment leads to platelet apoptosis through promoting oxidative stress in primary isolated platelets in an AT1R‐dependent manner.

### 3.7 Inhibiting the AT1R by losartan alleviates platelet apoptosis and thrombocytopenia in LPS‐induced endotoxemia mice by reducing oxidative stress and significantly increases the survival rate of LPS‐induced endotoxemia mice as well

**FIGURE 8:**
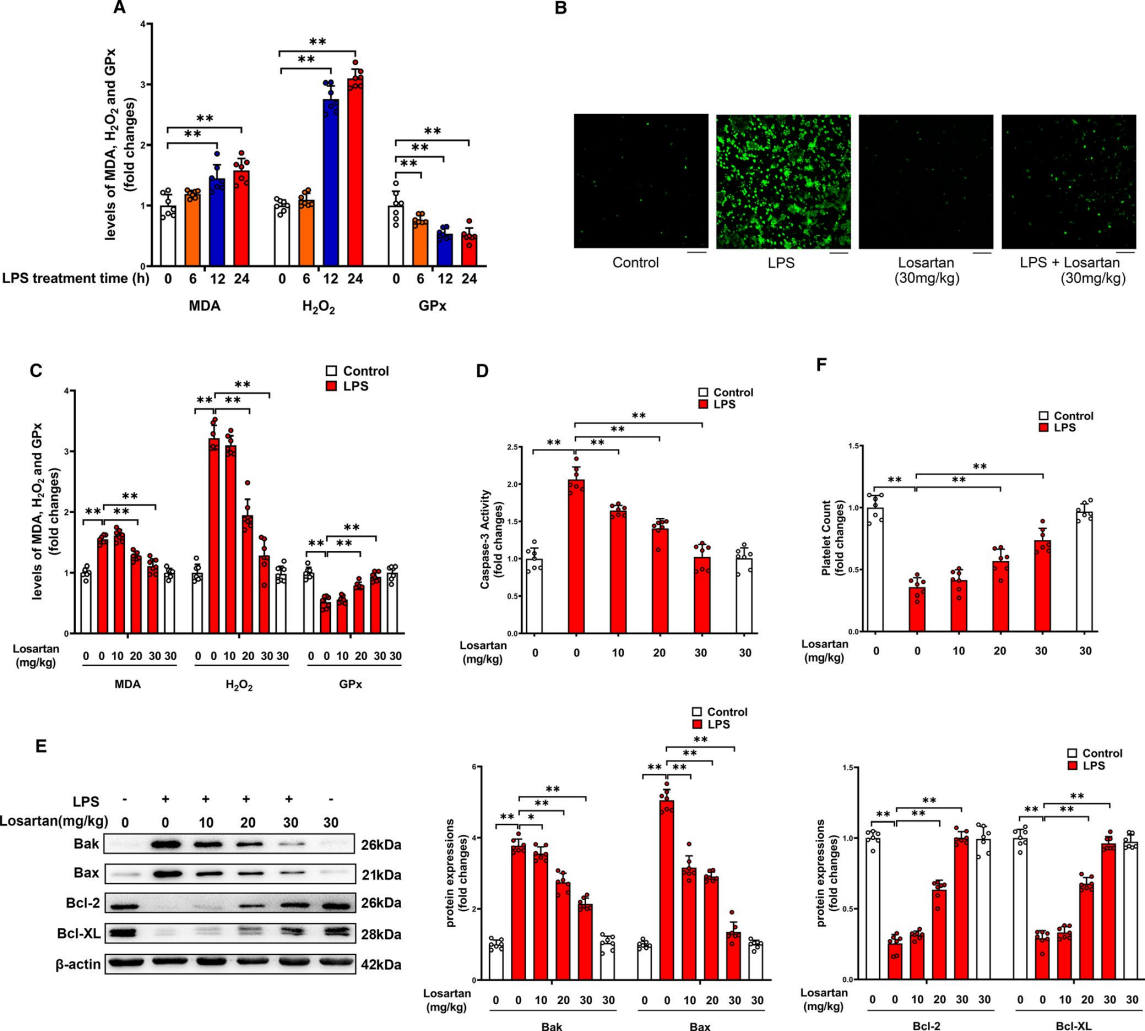
The effect of AT1R antagonist losartan on platelet oxidative stress, apoptosis and thrombocytopenia in LPS‐induced endotoxemia mice. Levels of MDA, H_2_O_2_ and GPx activity were determined in platelets (A) at the indicated time‐points (n = 7). AT1R antagonist losartan was administered at the indicated doses. 24 h later, ROS production (B), levels of MDA, H_2_O_2_ and GPx activity (C), caspase‐3 activity (D), expressions of Bak, Bax, Bcl‐2 and Bcl‐XL (E) in platelets, and platelet count (F) were determined (n = 7). Scale bars = 50 μm. *P < 0.05, **P < 0.01. kDa, kiloDalton

**FIGURE 9:**
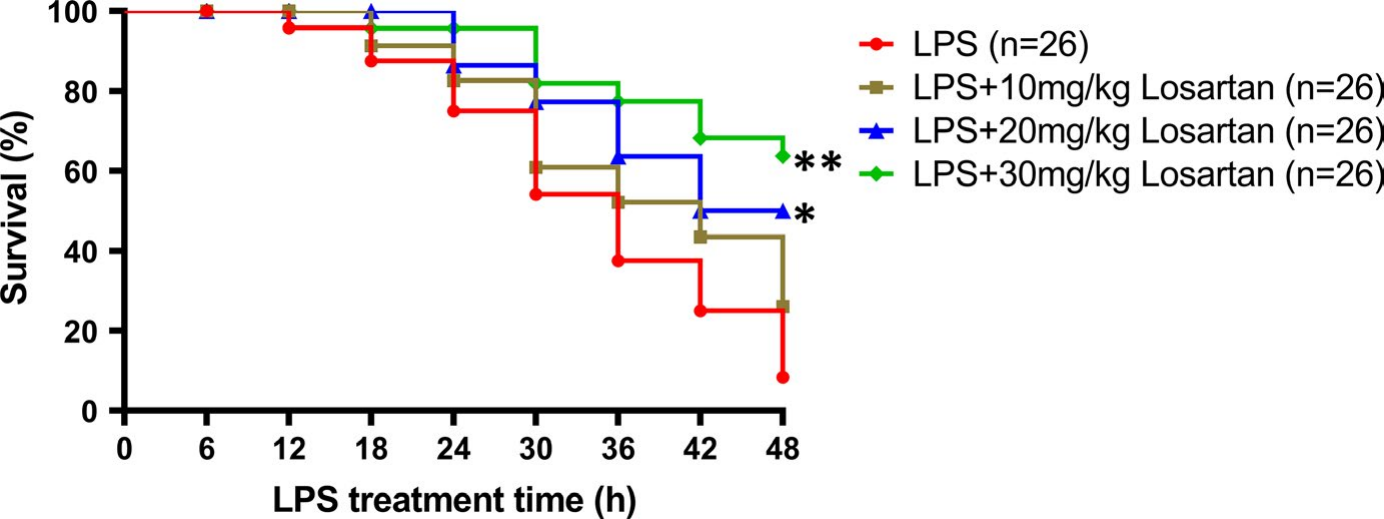
Losartan treatment significantly improves survival in LPS‐induced endotoxemia mice in concentration‐dependent manner. Survival rates of mice treated with saline (LPS group, n = 26) versus mice receiving losartan (10, 20 and 30 mg/kg, respectively) before LPS (LPS + Losartan group, n = 26) were compared. *P < 0.05, **P < 0.01

The effect of AT1R antagonist losartan on platelet oxidative stress, apoptosis and thrombocytopenia was then investigated in LPS‐induced endotoxemia mice. As expected, administration of LPS profoundly increased ROS production, MDA and H_2_O_2_ levels, whereas decreased GPx activity in platelets (Figure 8A). Losartan treatment (30 mg/kg) blocked LPS‐induced ROS production (Figure 8B). In addition, administration of losartan at the concentrations of 20 and 30 mg/kg dose‐dependently reduced platelet MDA and H_2_O_2_, whereas increased platelet GPx activity in LPS‐treated animals (Figure 8C). As shown in Figure 8D,E, administration of losartan at the concentrations of 10, 20 and 30 mg/kg reduced platelet caspase‐3 activity, and pro‐apoptotic protein levels (Bak and Bax), whereas increased anti‐apoptotic protein levels (Bcl‐2 and Bcl‐XL) in LPS‐treated animals in a dose‐dependent manner. Moreover, losartan dose‐dependently improved LPS‐induced thrombocytopenia (Figure 8F). We had analysed mice survival rate at 48 h after a lethal dose of LPS (30 mg/kg, i.p.) treatment with or without pre‐treatment with losartan (10, 20, 30 mg/kg, i.p.) to test the preventive therapeutic effect of losartan. As shown in Figure 9, only 4 of 26 mice survived in the group receiving saline‐only before the administration of LPS, while 9 of 26 mice pre‐treated with 10 mg/kg losartan, 15 of 26 mice pre‐treated with 20 mg/kg losartan and 18 of 26 mice pre‐treated with 30 mg/kg losartan survived in LPS‐treated group, indicating the protective effects of losartan in a concentration‐dependent manner during sepsis. Taken together, our findings indicated that AT1R antagonist losartan may alleviate platelet apoptosis and thrombocytopenia in LPS‐induced endotoxemia by reducing oxidative stress, thus significantly improve survival in LPS‐induced endotoxemia mice.

## 4 DISCUSSION

RAS activation as evidenced by increased PRA and Ang II is a well‐known phenomenon observed during the development of sepsis, both in experimental.^31^, ^32^ and clinical studies.^17^, ^33^, ^34^ A prospective cohort study of clinical sepsis has shown that plasma Ang II is elevated in septic patients as compared to volunteers, and the degree of Ang II elevation correlates with organ failure and with measures of microvascular dysregulation.^17^ Huang et al report that plasma Ang II concentrations are associated with disease severity induced by H7N9 influenza virus and may potentially predict patient mortality.^35^ The present study revealed for the first time that elevated plasma Ang II levels were linked to the severity of thrombocytopenia both in septic patients and in LPS‐induced endotoxemia mice. These initial observations indicate that RAS activation may be involved in the pathogenesis of sepsis‐associated thrombocytopenia.

Platelets play critical roles in the development of sepsis. Accumulating studies have recognized thrombocytopenia as a strong predictor of mortality in sepsis and other infectious diseases.^11^, ^36^, ^37^, ^38^ A primary cause of sepsis‐associated thrombocytopenia is shortened survival of platelets.^9^ Previous studies have shown that the septic milieu impairs mitochondrial function, and decreases the expression of the anti‐apoptotic protein Bcl‐XL, which is required for the maintenance of platelet survival.^11^, ^37^ In addition, both gram‐negative and gram‐positive bacteria can induce Bcl‐XL protein degradation in platelets.^11^ Pathogenic bacteria can directly activate the intrinsic apoptotic pathway to induce platelet cell death in vitro.^10^, ^11^ By using primary isolated platelets, the present study showed that Ang II treatment directly stimulated platelet apoptosis in an AT1R‐dependent manner in vitro. In addition, the AT1R antagonist losartan profoundly reversed LPS‐induced thrombocytopenia in mice. These findings provide the first evidence that elevated Ang II in septic milieu can directly induces apoptotic cell death in platelets, which represents a novel mechanism of sepsis‐induced thrombocytopenia.

As an important pro‐oxidative and pro‐inflammatory agent, Ang II has been implicated in organ failure and mortality during infectious diseases.^39^, ^40^, ^41^ Ang II also leads to an up‐regulation of tissue factor, an important component of thrombogenesis and subsequent vascular dysfunction and microvascular ischaemia in sepsis.^42^, ^43^ In animal models of sepsis, therapies directed against Ang II are associated with lower levels of pro‐inflammatory cytokine and oxidative stress, improved endothelial function and improved survival.^44^, ^45^, ^46^, ^47^ It is noteworthy that in the past two years, several population‐based retrospective cohort studies indicate that prior use of ARBs is associated with decreased short‐term mortality after sepsis.^48^, ^49^, ^50^, ^51^ In particular, Hsieh et al recently report that regardless of a non‐shock or septic shock condition, decreased risk of total hospital mortality is associated with preadmission ARB use.^48^ Thrombocytopenia has been recognized as a strong predictor of mortality in sepsis.^2^ The present study demonstrated that blockade of AT1R significantly improved sepsis‐associated thrombocytopenia. Our findings provided a potential explanation for the clinical benefits of pre‐hospitalization use of ARBs in patients with sepsis.

In summary, our data suggest that elevated plasma Ang II is associated with thrombocytopenia in both septic patients and LPS‐induced endotoxemia mice. Using primary isolated platelets, Ang II directly stimulated platelet apoptosis through promoting oxidative stress in an AT1R‐dependent manner. Additionally, in vivo experiments evidenced the protective effects of AT1R antagonist losartan against platelet apoptosis and thrombocytopenia induced by LPS treatment. The present study identifies a novel function of elevated Ang II in sepsis‐associated platelet apoptosis. Antagonist targeting AT1R might have clinical benefit in alleviating sepsis‐associated thrombocytopenia.

## CONFLICT OF INTEREST

The authors have no conflict of interest to declare.

## AUTHOR CONTRIBUTIONS

Dun‐Feng Xu: Data curation (equal); Formal analysis (equal); Methodology (equal); Software (equal); Writing‐original draft (lead). Yu Jian Liu: Formal analysis (equal); Funding acquisition (equal); Methodology (equal). Yan Fei Mao: Funding acquisition (equal); Investigation (equal). Yan Wang: Methodology (supporting). Chu Fan Xu: Methodology (supporting). Xiao‐Yan Zhu: Funding acquisition (equal); Methodology (equal); Supervision (equal); Writing‐review & editing (equal). Lai Jiang: Funding acquisition (equal); Project administration (equal); Supervision (equal); Visualization (equal).

## Supporting information

Supplementary Material

## Data Availability

The datasets supporting the conclusions of this study are available from the corresponding author upon request.
