# Supplementary material for: Elevated angiotensin II induces platelet apoptosis through promoting oxidative stress in an AT1R‐dependent manner during sepsis
Source: J Cell Mol Med. 2021 Feb 23;25(8):4124–35. doi: 10.1111/jcmm.16382 (PMC8051711; doi:10.1111/jcmm.16382)
Supplement: Supplementary file 1 — Supplementary Material [file JCMM-25-4124-s001.docx]

**Elevated angiotensin II induces platelet apoptosis through promoting oxidative stress in an AT1R-dependent manner during sepsis**

**Dun-Feng Xu, PhD^1,2,a^, Yu-Jian Liu, PhD^2,a^, Yan-Fei Mao, PhD^1^, Yan Wang, MD^1^, Chu-Fan Xu, PhD^1,2^, Xiao-Yan Zhu, PhD ^3,*^, Lai Jiang, PhD ^1,*^**

^1^Department of Anesthesiology and Surgical Intensive Care Unit, Xinhua Hospital, Shanghai Jiao Tong University School of Medicine, Shanghai 200092; ^2^School of Kinesiology, The key Laboratory of Exercise and Health Sciences of Ministry of Education, Shanghai University of Sport, Shanghai 200438; ^3^Department of Physiology, Navy Medical University, Shanghai 200433

**Supplemental Methods**

**Plasma of healthy volunteers and patients with sepsis**

The research protocol received approval from the Ethics Committee of Xinhua Hospital Affiliated to Shanghai Jiao Tong University School of Medicine, and written informed consents were obtained from all patients. This research project has registered its clinical trial in ClinicalTrails.gov (Clinicaltrials.gov NCTNCT03952390; https://clinicaltrials.gov/ct2/show/ NCT03952390). All of the septic patients were recruited from the Intensive Care Unit (ICU) of Xinhua Hospital from January 2018 to June 2019. Healthy volunteers were randomly recruited from healthy adults who underwent routine physical examinations. Sepsis was defined according to the 2016 SCCM/ESICM/ACCP/ATS/SIS International Sepsis Definitions Conference.^1^ Inclusion criteria included: 1) Diagnosis or clinical diagnosis of infection; 2) The change of Sequential Organ Failure Assessment (ΔSOFA) ≥ 2; 3) Informed consent. Exclusion criteria included: 1) Pregnant or lactation period; 2) Age <18 years or >85 years; 3) Receiving chemotherapy, steroid or immunosuppressive agents recently; 4) Receiving any drugs that affect renin-angiotensin system (RAS), such as angiotensin converting enzyme inhibitor (ACEI), angiotensin receptor blocker (ARB), diuretics, calcium channel blockers and other antihypertensive within two weeks; 5) Receiving oral contraceptives within twelve weeks; 6) Resuscitation before enrollment. Blood samples were collected in tubes with an inhibitor cocktail (EDTA, dimercaprol and 8-hydroxyquinoline sulfate) to prevent generation or degeneration of angiotensin II (Ang II) ex vivo, and were centrifuged immediately at 2500 rpm for 10 minutes at 4°C. Plasma was frozen and stored at −80°C until assayed.

**Endotoxemia model and drug treatment**

Male Institute of Cancer Research (ICR) mice (7~9-weeks-old) were obtained from Shanghai SLAC Laboratory Animal Co. (Shanghai, China) and housed at controlled room temperature with free access to food and water under a natural day/night cycle. All animal protocols were approved by the Ethics Committee on Experimental Animals of Shanghai Jiao Tong University School of Medicine. Puriﬁed lipopolysaccharide (LPS) extracted from the membrane of Escherichia coli 0111:B4 (Sigma-Aldrich, St. Louis, MO) was dissolved in sterile pyrogen-free saline and injected i.p. at a dose of 5 mg/kg.^2^ Losartan (Selleck, Houston, TX), the antagonist for angiotensin II type 1 receptor (AT1R) dissolved in sterile saline, was injected i.p. 30 minutes prior to the injection of LPS at a dose of 10~30 mg/kg. N-acetyl-l-cysteine (NAC), a reactive oxygen species (ROS) scavenger dissolved in sterile saline, was injected i.p. 30 minutes prior to the injection of LPS at a dose of 100 mg/kg. The control group received an equivalent volume of saline. Blood samples were collected in tubes with an inhibitor cocktail (EDTA, dimercaprol and 8-hydroxyquinoline sulfate) to prevent generation or degeneration of Ang II ex vivo, and were centrifuged immediately at 2500 rpm for 10 minutes at 4°C. Plasma was frozen and stored at −80°C until assayed.

**Measurements of plasma renin activity (PRA) and angiotensin II (Ang II) concentration**

Radioimmunoassay kit (Beijing North Institute of Biological Technology, Beijing, China) was used to check the PRA and Ang II concentration in Department of Radioimmunoassay and Clinical Laboratory (Luwan Branch of Ruijin Hospital, Shanghai Jiao Tong University School of Medicine). The renin activity in the plasma was assessed by measuring their ability to generate angiotensin I (Ang I) from angiotensinogen.^3^ The results were expressed as picograms of Ang I per millilitre of plasma per hour of incubation.

**Mouse platelet isolation and treatment**

Mouse platelets were isolated using a modified method as previously described.^4, 5^ Briefly, after anesthesia with a mixture of ketamine (70 mg/kg) and xylazine (10 mg/kg), blood was collected from the right ventricle with 1:10 acid citrate dextrose anticoagulant and immediately diluted with equal volumes of modified Tyrode solution (1 µg/mL prostaglandin E1 and 0.1 U/mL apyrase), centrifuged at 200 g for 5 minutes. The resulting platelet-rich plasma (PRP) was collected and centrifuged at 800 g for 15 minutes. Platelet pellets were washed twice and resuspended in Tyrode solution at a count of 5*10^^7^ platelets per tube.

**Western blot analysis**

The washed platelets were homogenized in RIPA lysis buffer (Beyotime, Jiangsu, China) containing 1% Proteinase Inhibitor Cocktail (Sigma-Aldrich) at 4°C. 30 ug of protein were separated by 12% sodium dodecyl sulfate polyacrylamide gel electrophoresis (SDS-PAGE) and subsequently transferred to nitrocellulose membranes (Millipore Corp, Bedford, MA). After blocking, immunoblots were incubated with primary antibody against Bax (Abcam, Cambridge, UK), Bcl-2 (Abcam), Bcl-XL (Abcam), Bak (Cell Signaling Technology, Beverly, MA), AT1R (Santa Cruz Biotechnology, Santa Cruz, CA), and AT2R (Abcam) at 4°C overnight. The membrane was incubated with the secondary horseradish peroxidase (HRP)-conjugated antibodies for 1 hour at room temperature followed by several washes. Immunoreactive proteins were visualized using the enhanced chemiluminescence Western blotting detection system (Millipore). To control sampling errors, each normalized control was expressed as the ratio of protein of interest to the loading control β-actin.

**Measurement of Caspase-3 colormetric proteolytic activity**

Caspase-3 activity was determined by using the Caspase 3 Activity Assay Kit (Beyotime, Jiangsu, China).^6^ Briefly, platelets were washed and lysed in 100 μl lysis buffer and cell debris were removed by centrifugation. 50 μl of clariﬁed supernatants were added to 50 μl of assay buffer and 10 μl of the 2 mM Ac-DEVD-pNA chromogenic substrate into 96-well plates with 50 μl of lysis buffer served as control. After incubating for 2 hours at 37°C at dark, the absorbance of the reaction product pNA was read at 405 nm. The absorbance was transferred to pNA concentration according to the standard curve. Caspase-3 activity was expressed as normalized pNA concentration over total protein content.

**Detection of ROS**

Intracellular ROS levels were measured with fluorescence probe 2',7'-dichlorofluorescein diacetate (DCFH-DA, Sigma-Aldrich) as previously described.^7^ Briefly, the washed platelets were settled on glass-bottom dishes for 30 minutes at 37°C, then incubated with DCFH-DA (10 μM) for 20 minutes at 37°C and washed twice with PBS. Fluorescence images were taken with a confocal laser scanning microscopy (LSM700; Carl Zeiss Co., Germany) and the intensity of fluorescence was analyzed by Image Pro Plus software.

**Detection of malondialdehyde (MDA)**

Levels of MDA, as an index of membrane lipid peroxidation, were determined as previously described.^8^ Platelets were homogenized (100 mg/mL) in 10 vol of 1.15% KCl solution containing 0.85% NaCl and then centrifuged at 1500 g for 15 minutes. 200 μl of the homogenates were then added to a reaction mixture consisting of 1.5 ml 0.8% thiobarbituric acid, 200 μl 8.1% sodium dodecyl sulfate, 1.5 ml 20% acetic acid (adjusted to pH 3.5 with NaOH) and 600 μl distilled H_2_O. The mixture was then heated at 95 °C for 40 minutes. After cooling to room temperature, the samples were cleared by centrifugation (10,000 g, 10 min) and their absorbance measured at 532 nm, using 1,1,3,3-tetramethoxypropane as an external standard. The levels of lipid peroxides were expressed as nmol MDA/mg protein (Bradford assay).

**Supplementary References**

1.Singer M, Deutschman C S, Seymour C W, et al. The Third International Consensus Definitions for Sepsis and Septic Shock (Sepsis-3). JAMA. 2016; 315(8): 801-810.DOI: 10.1001/jama.2016.0287

2.Wang C N, Liu Y J, Duan G L, et al. CBS and CSE are critical for maintenance of mitochondrial function and glucocorticoid production in adrenal cortex. Antioxid Redox Signal. 2014; 21(16): 2192-2207.DOI: 10.1089/ars.2013.5682

3.Wu Y, Ma K L, Zhang Y, et al. Lipid disorder and intrahepatic renin-angiotensin system activation synergistically contribute to non-alcoholic fatty liver disease. Liver Int. 2016; 36(10): 1525-1534.DOI: 10.1111/liv.13131

4.Wang Y, Zhang S, Luo L, et al. Platelet-derived microparticles regulates thrombin generation via phophatidylserine in abdominal sepsis. J Cell Physiol. 2018; 233(2): 1051-1060.DOI: 10.1002/jcp.25959

5.Wrzyszcz A, Urbaniak J, Sapa A, et al. An efficient method for isolation of representative and contamination-free population of blood platelets for proteomic studies. Platelets. 2017; 28(1): 43-53.DOI: 10.1080/09537104.2016.1209478

6.Xu C F, Liu Y J, Wang Y, et al. Downregulation of R-Spondin1 Contributes to Mechanical Stretch-Induced Lung Injury. Crit Care Med. 2019; 47(7): e587-e596.DOI: 10.1097/CCM.0000000000003767

7.Lv Z, Wang Y, Liu Y-J, et al. NLRP3 Inflammasome Activation Contributes to Mechanical Stretch–Induced Endothelial-Mesenchymal Transition and Pulmonary Fibrosis. Critical Care Medicine. 2018; 46(1): e49-e58.DOI: 10.1097/ccm.0000000000002799

8.Zhang Y-Q, Liu Y-J, Mao Y-F, et al. Resveratrol ameliorates lipopolysaccharide-induced epithelial mesenchymal transition and pulmonary fibrosis through suppression of oxidative stress and transforming growth factor-β1 signaling. Clinical Nutrition. 2015; 34(4): 752-760.DOI: 10.1016/j.clnu.2014.08.014
